# Dual-Tracer Imaging on a Long–Axial-Field-of-View PET: A Proof-of-Principle Study with [^18^F]FGln and [^18^F]FDG

**DOI:** 10.2967/jnumed.124.268831

**Published:** 2025-07

**Authors:** Daniel Kwon, Elizabeth J. Li, Christina Dulal, Margaret Daube-Witherspoon, Varsha Viswanath, Anthony J. Young, Raheema A. Damani, Jason Hou-Liu, Elizabeth S. McDonald, David A. Mankoff, Joel S. Karp, Austin R. Pantel

**Affiliations:** 1 Department of Radiology, University of Pennsylvania, Philadelphia, Pennsylvania;; 2 MD/PhD Training Program, Faculty of Medicine, University of British Columbia, Vancouver, British Columbia, Canada; and; 3 Department of Statistics and Actuarial Science, University of Waterloo, Waterloo, Ontario, Canada

**Keywords:** PET, long–axial-field-of-view PET/CT, cancer metabolism, dynamic PET imaging, dual-tracer PET

## Abstract

Current protocols necessitate imaging 2 ^18^F-labeled tracers in separate sessions. Herein, we report on the development and testing of a protocol that sequentially images 2 ^18^F-labeled tracers—^18^F-(2*S,*4*R*)4-fluoroglutamine, commonly known as [^18^F]FGln, and [^18^F]FDG—in a single session on a long–axial-field-of-view PET scanner to study cancer metabolism. **Methods:** A single [^18^F]FGln scan was used to estimate the minimal injected activity and scan duration to calculate an accurate volume of distribution. This was followed by a dual-tracer study in a second patient with a low-dose (41-MBq), shortened (29-min) [^18^F]FGln scan, followed by a full-dose (394-MBq) [^18^F]FDG scan for 60 min. Protocol performance was assessed using the resulting kinetic parameters. **Results:** [^18^F]FGln subsampling demonstrated stable estimates of volume of distribution with a scan duration of 30 min and a dose of 37 MBq. The dual-tracer [^18^F]FDG image obtained 60 min after injection was of diagnostic quality, with minimal (6%) residual [^18^F]FGln signal. **Conclusion:** This proof-of-principle dual-tracer study demonstrated the feasibility of a [^18^F]FGln/[^18^F]FDG protocol that leveraged the high sensitivity of long–axial-field-of-view PET to inject the first tracer at a low dose and the second tracer at a greater dose, overwhelming the signal from the first tracer.

## Visual Abstract

**Figure:**
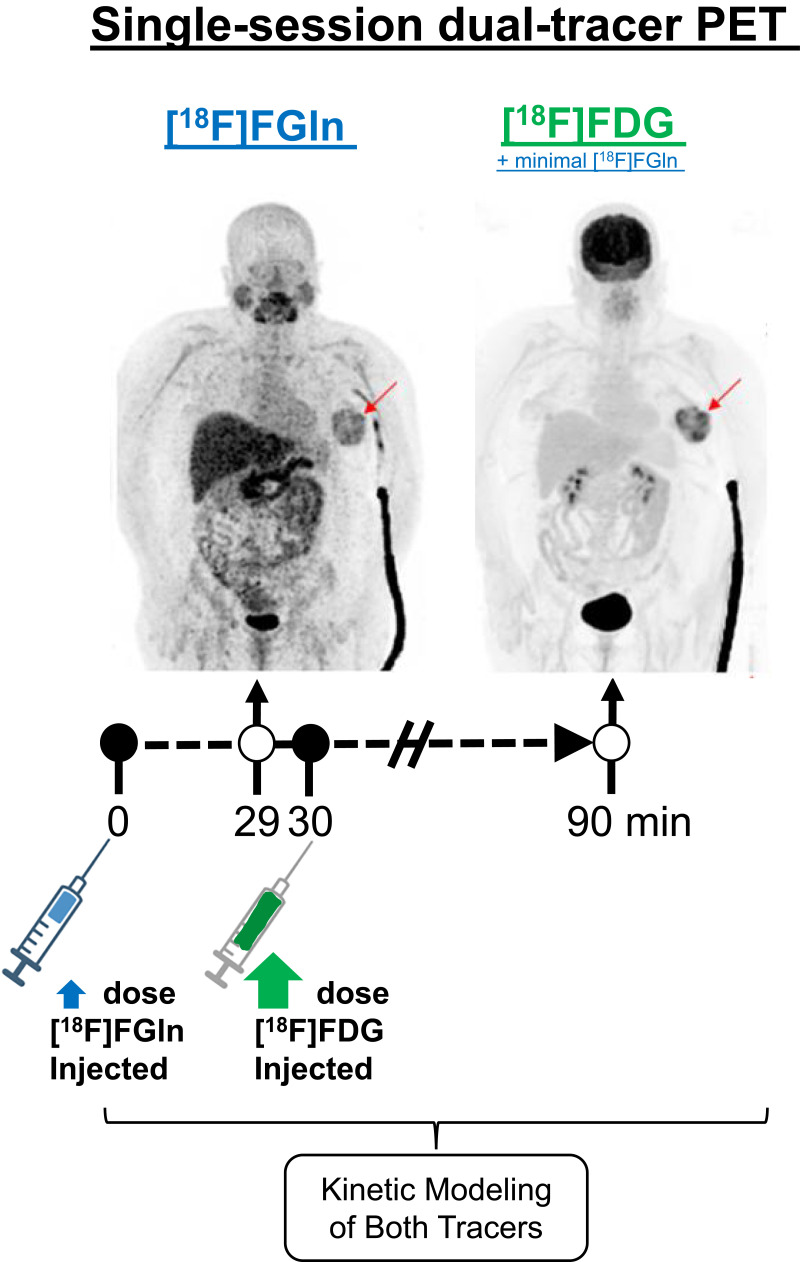

Numerous PET radiotracers have recently been developed and integrated into clinical practice, advancing precision medicine through molecular imaging (1*–*3). However, routine PET protocols cannot image multiple long-lived PET tracers (e.g., ^68^Ga and ^18^F) in the same imaging session, as the first tracer must decay before the second can be administered. This necessitates a separate imaging session on a different day, duplicating efforts (e.g., patient preparation, CT scans), burdening sick patients, and delaying a comprehensive assessment of disease. Moreover, inherent differences in patient position (and tumor biology) between the 2 scans create interpretation challenges for the radiologist and hamper direct application of advanced analytic methods. The development of protocols that enable the imaging of multiple PET radiotracers in a single scanning session would benefit PET imaging, with both clinical and research applications.

Long–axial-field-of-view (LAFOV) PET (4) produces superb images with substantially lower injected activity relative to modern short–axial-field-of-view PET scanners (typical axial fields of view of <30 cm), creating opportunities for the development of innovative protocols. We developed a dual-tracer protocol with 1 patient and tested the protocol with another patient using the LAFOV PennPET Explorer (5) to image 2 ^18^F-labeled tracers sequentially in the same imaging session (4): first with a low injected activity of ^18^F-(2*S,*4*R*)4-fluoroglutamine ([^18^F]FGln), followed by a second tracer, [^18^F]FDG, with a greater injected activity (Fig. 1). By near-simultaneous measurement of tumoral glutaminolysis with [^18^F]FGln (6*,*^7^) and tumoral glycolysis with [^18^F]FDG (8), we aim to better characterize the metabolic phenotype of cancer. This paradigm (low injected activity, followed by high injected activity) uses LAFOV PET to derive kinetic parameters from both tracers to characterize disease and differs from protocols used in other studies of dual tracers, including those performed with conventional scanners using typical doses, which allowed for quantification of both tracers (3′-deoxy-3′-^18^F-fluorothymidine and [^18^F]FDG) using sophisticated kinetic analysis methods (9), as well as prior static studies on LAFOV scanners without kinetic quantification (10), including a study using low doses of 2 tracers (11). This study provides proof-of-concept of a novel imaging paradigm.

**FIGURE 1.**
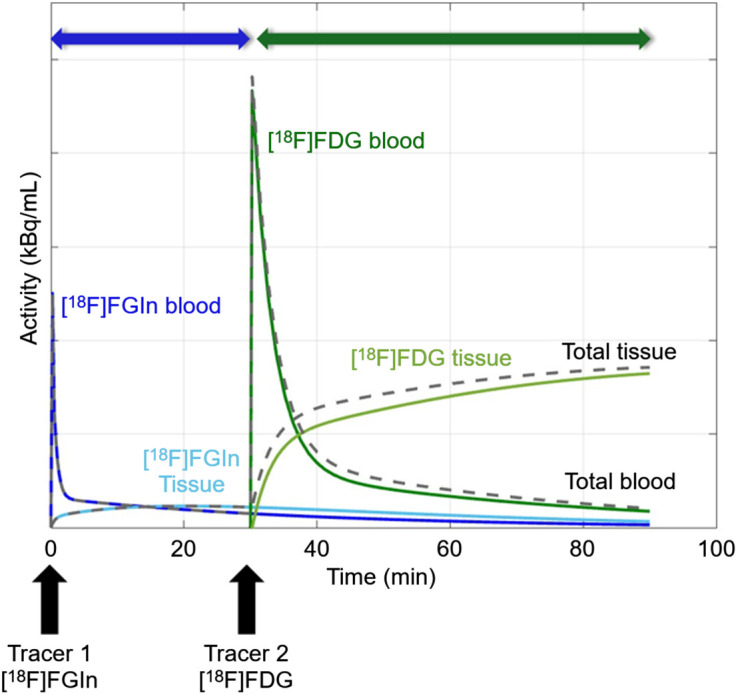
Proposed dual-tracer study schema.

## MATERIALS AND METHODS

### Clinical Trial

Both patients studied were enrolled in the [^18^F]F-GLN by PET/CT in Breast Cancer (NCT03863457) clinical trial, approved by the University of Pennsylvania Institutional Review Board. All patients gave written informed consent. Patients age 18 y or older with known or suspected primary or metastatic breast cancer with at least 1 lesion of 1.5 cm or greater in diameter (measured by standard-of-care imaging) were eligible for inclusion.

### Single-Tracer [^18^F]FGln Imaging Protocol

**FIGURE 2.**
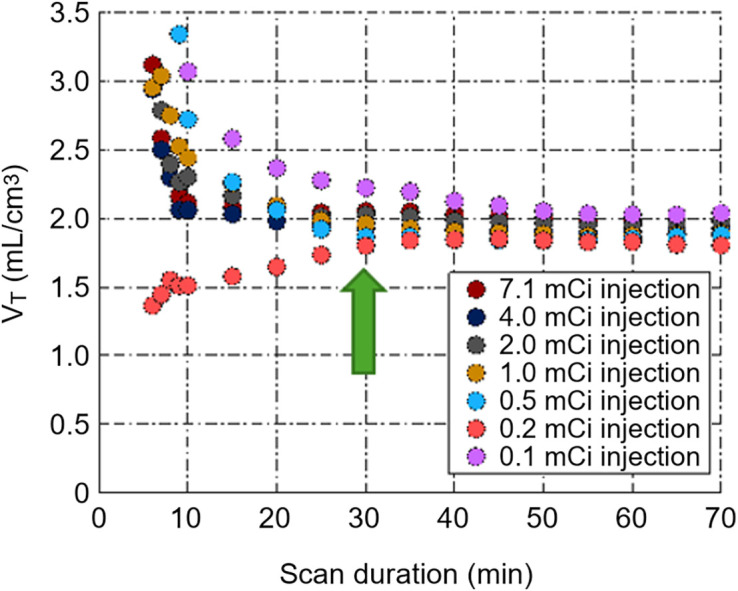
Subsampling studies of a dynamic [^18^F]FGln study. Full-dose [^18^F]FGln study (263 MBq [7.1 mCi]; scan duration of 70 min) was subsampled to estimate lowest possible dose and scan time (green arrow) while faithfully recapitulating calculation of *V*_T_ compared with full dataset.

[^18^F]FGln was synthesized as previously described (12). An 84.5-kg patient with breast cancer was injected with full-dose (263-MBq [7.1-mCi]) [^18^F]FGln and dynamically scanned for 70 min on the PennPET Explorer (5*,*^13^*,*^14^). List-mode data were subsampled to emulate 6 doses (3.7–148 MBq [0.1–4 mCi]) and to determine the minimal activity and scan duration needed to recover the volume of distribution (*V*_T_), the kinetic parameter of interest (Fig. 2).

### Dual-Tracer [^18^F]FGln/[^18^F]FDG Imaging Protocol

The single-tracer study was used to design the protocol (i.e., dose and scan duration) for the [^18^F]FGln/[^18^F]FDG dual-tracer study. A second patient, weighing 93.5 kg, with estrogen receptor–positive/progesterone receptor–negative/human epidermal growth factor receptor–negative breast cancer was imaged with 41 MBq (1.1 mCi) of [^18^F]FGln for 29 min and then with 394 MBq (10.6 mCi) of [^18^F]FDG for an additional 60 min on the PennPET Explorer (5). Image reconstruction details are provided in the supplemental materials, available at http://jnm.snmjournals.org. To assess the stability of the *V*_T_ estimate, the [^18^F]FGln data (0–29 min) were subsampled without replacement to half and quarter doses (20.4 and 10.4 MBq [0.55 and 0.28 mCi], respectively).

### Image Analysis

PET/CT images were viewed with MIM software, version 7.1.5, and PMOD, version 3.7 (PMOD Technologies Ltd.). Volumes of interest (VOIs) were placed over the whole tumor, whole gray matter, left and right kidneys, myocardium, right quadricep, and spleen. Another VOI (9 cm^3^) was placed over the descending aorta to serve as an image-derived input function.

### Kinetic Analysis

Two types of modeling software were used. Kinetic analysis was performed using PMOD and mfEVolve, version 1.6 (MultiFunctional Imaging LLC). PMOD, a widely used image analysis software package, was used to investigate [^18^F]FDG modeling by subtracting the residual [^18^F]FGln signal, with the intent of providing an analysis tool familiar to many users. mfEVolve, a software package specifically designed to analyze dual-tracer studies that optimize kinetic parameter estimates for each tracer simultaneously, was used as a comparator to PMOD in combination with simpler subtraction methods (15*,*^16^). mfEVolve leverages separable parameter space kinetic modeling (a reformulation of traditional compartment modeling methods) in which the number of linear parameters is maximized and the number of nonlinear parameters is minimized. This reduces the dimensionality and improves the speed of the fitting process, as the linear terms can be determined directly for each nonlinear fit (which is then determined by either an exhaustive search or use of the Levenberg-Marquardt fitting algorithm).

For the tumor, a 1-tissue-compartment model was used for [^18^F]FGln on the basis of prior work in preclinical models (6) and prior human studies described in this article. A 2-tissue-compartment model with irreversible trapping (FDG dephosphorylation rate constant, 0) was used for [^18^F]FDG (8). The Levenberg-Marquardt fitting algorithm was used in both PMOD and mfEVolve to fit the compartment models. Logan and Patlak plots were constructed with PMOD to calculate the [^18^F]FGln *V*_T _and [^18^F]FDG net influx rate (*K*_i_), respectively. Fitting details for other organs are included in supplemental materials. Blood fraction and delay were included in the fitting process for all tissues.

### Correction for [^18^F]FGln Signal After [^18^F]FDG Injection

After removing decay correction from the dual-tracer study, the [^18^F]FDG image-derived blood input function was corrected for the [^18^F]FGln signal by subtracting the projected [^18^F]FGln input function curve, based on a fit to a triexponential function. After fitting the [^18^F]FGln tumor and healthy tissue curves, we used the estimated kinetic parameters and projected input function to project the [^18^F]FGln tissue curves in PMOD, which were then subtracted from the [^18^F]FDG portion of the study. Decay correction was then reapplied before final fitting of the [^18^F]FGln and [^18^F]FDG portions of the study.

## RESULTS

### Single-Tracer [^18^F]FGln PET to Determine Scan Parameters

Subsampling studies, guided by prior preclinical work (6), demonstrated that short scan durations and low doses led to instabilities in *V*_T_ estimates (Fig. 2). A 37-MBq (1-mCi) dose of [^18^F]FGln injection and a scan duration of 30 min were selected for the proposed study because the *V*_T_ was similar to the reference (full dose and 70-min scan duration), whereas shorter scans with less injected activity deviated from the reference *V*_T_.

### Dual-Tracer [^18^F]FGln/[^18^F]FDG Study

**FIGURE 3.**
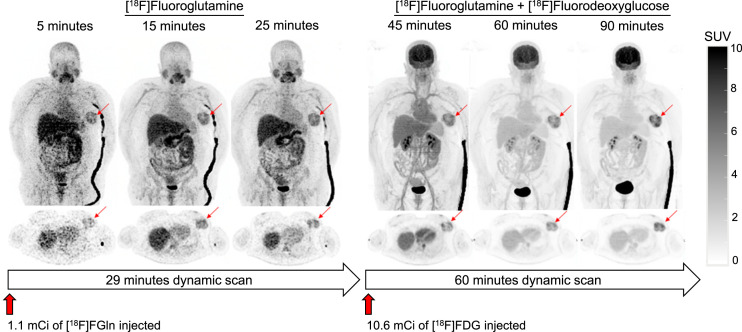
Representative images from dual-tracer dynamic study. Maximum-intensity-projection PET images of [^18^F]FGln (left) and [^18^F]FGln/[^18^F]FDG (right). [^18^F]FDG was injected at 29 min.

Dynamic [^18^F]FGln PET imaging demonstrated the expected distribution of the tracer (Fig. 3). The final [^18^F]FGln PET image (taken 25–29 min after injection) was of diagnostic quality. Similarly, dynamic [^18^F]FDG imaging (with residual [^18^F]FGln present) demonstrated the expected distribution of [^18^F]FDG without obvious confounding from the [^18^F]FGln signal. Uptake of both tracers correlated with the breast cancer seen on anatomic imaging (Supplemental Fig. 1).

### Kinetic Analysis of [^18^F]FGln Dynamic PET

**FIGURE 4.**
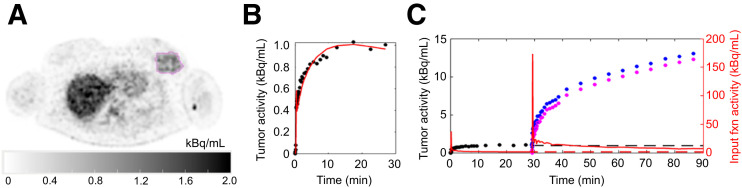
Representative axial [^18^F]FGln image (A) and whole-tumor time–activity curve (red) with VOI measurements (black) (B). (C) [^18^F]FGln input function (red) and tumor (black) time–activity curves, projected [^18^F]FGln values (dotted lines), [^18^F]FGln/[^18^F]FDG values (blue), and [^18^F]FDG values with [^18^F]FGln signal correction (magenta).

[^18^F]FGln demonstrated an SUV_mean _of 2.2 and SUV_max _of 6.2 of the tumor VOI (193 mL volume) at 25 min after injection (4-min frame; Fig. 4). One-tissue-compartment–derived *V*_T _values of 0.60 and 0.58 mL/cm^3 ^were calculated using PMOD and mfEVolve, respectively (Supplemental Table 1). The Logan plot *V*_T_ was 0.65 mL/cm^3^ (Supplemental Fig. 2). Compartment modeling–based *V*_T_ values for other tissues showed differences of less than 15% for most tissues (Supplemental Table 2). The [^18^F]FGln images from subsampled data (half and quarter doses) demonstrated increased noise (Supplemental Fig. 3), and 1-tissue-compartment–derived tumor *V*_T_ values (via PMOD) were higher with subsampling (half dose, 0.95; quarter dose, 1.00).

### [^18^F]FDG and Residual [^18^F]FGln Signals

The [^18^F]FDG SUV_mean_ in the final 5-min frame (55 min after injection, akin to a delayed static image obtained routinely in the clinic) with and without [^18^F]FGln signal correction showed a difference of 6% (SUV_mean _of 2.99 and 3.18, respectively) (Fig. 4C).

### Kinetic Analysis of [^18^F]FDG

The [^18^F]FDG *K*_i_, without adjusting for the residual [^18^F]FGln signal, was 0.0144 mL/min/cm^3^, with a Patlak-based estimate of 0.0146 mL/min/cm^3^ (Supplemental Tables 2 and 3). With [^18^F]FGln signal correction, whole-tumor *K*_i_ increased to 0.0157 mL/min/cm^3^ (difference of 9%). mfEVolve’s multitracer methodology showed agreement, with a *K*_i_ of 0.0157 mL/min/cm^3^. Gray matter showed the largest absolute difference in *K*_i_ across software (0.0053 mL/min/cm^3^; difference of 7.8%). Large differences (>10%) were observed when correcting for residual [^18^F]FGln signal in the right kidney, myocardium, and spleen (Supplemental Table 2).

## DISCUSSION

We developed and performed initial validation of a sequential (same-session) dual-tracer dynamic PET protocol for 2 ^18^F-labeled radiotracers. We leveraged the high sensitivity of LAFOV PET to inject the first tracer at a low dose and the second at a greater dose, overwhelming the signal from the first tracer. The PennPET Explorer produced diagnostic-quality images, without noticeable visual confounding from the [^18^F]FGln signal after [^18^F]FDG administration. We found only a 6% contribution of [^18^F]FGln in the last frame of the [^18^F]FDG study, which likely can be ignored in practice (17*–*20). Similarly, whole-tumor [^18^F]FDG *K*_i_ was only 9% lower without accounting for residual [^18^F]FGln. Work is under way to study the effects on smaller VOIs (e.g., 1-cm^3^ VOIs) (17). [^18^F]FGln signal correction of the tumor using PMOD yielded results similar to those found with mfEVolve. However, there were some relatively large (>20%) differences in *K*_i_ across regions, particularly for highly perfused tissues (e.g., myocardium, kidneys) that will be investigated further with additional patients. Use of the Levenberg-Marquardt fitting algorithm, where local minima can occur, or continuous-to-discrete integration models between PMOD and mfEVolve may contribute to differences between these software packages. Additional applications, such as in lesions with high uptake of the first tracer, may necessitate using mfEVolve, where its specialized workflow and computational efficiency may be of more benefit.

The estrogen receptor–positive tumor demonstrated modest [^18^F]FGln uptake, reflecting a relatively large glutamine pool, and moderate [^18^F]FDG uptake, consistent with the expected biology, given this molecular phenotype (7*,*^21^). The ability to image 2 facets of metabolism—glycolysis and glutaminolysis—in succession advances our ability to characterize tumoral biology. Moreover, inherent spatial registration between scans will facilitate advanced image analysis methods, including heterogeneity analysis and radiomics (22*,*^23^).

This proof-of-concept, single-subject study had several limitations. First, the protocol for each unique dual-tracer pair must be individually designed and validated for statistical robustness, so the scan parameters used here are not directly applicable to all tracer pairs or subjects. The current study did not have reference standards for the kinetic parameters of interest, so the accuracy of low-dose [^18^F]FGln *V*_T_ was inferred via subsampling. Moreover, this study did not account for radiometabolites or blood-to-plasma partitioning of [^18^F]FGln, with our prior work in mice demonstrating only a small fraction of metabolites (≤16%) (7); in our patient, a single venous blood sample taken approximately 30 min after [^18^F]FGln injection had a measured parent fraction of 97%. Future publications with more patients will account for metabolite data collected via venous blood sampling. Lastly, the increased sensitivity of the PennPET Explorer may not directly predict performance in standard axial-field-of-view scanners. Additional subsampling studies are underway to guide the translation of the dual-tracer protocols to SAFOV scanners (24).

Dual-tracer approaches have been studied before, including fully quantitative approaches with standard axial-field-of-view PET as well as more qualitative approaches using LAFOV PET. Research protocols leveraging differences in radioisotope properties, including γ-emissions and half-lives, have been initiated but are far from translation into clinical practice. This protocol minimally increases radiation exposure compared with a single PET scan (with opportunity to further decrease the dose) and provides additional comparative benefits. The protocol developed here, which may obviate the use of advanced modeling approaches in select cases while still allowing for kinetic modeling of 2 tracers, has imminent translational potential, with widespread adoption facilitated by imaging centers acquiring commercial LAFOV PET and imaging multiple tracers.

## CONCLUSION

A sequential (same-session) dual-tracer dynamic PET protocol for 2 ^18^F-labeled radiotracers was performed in this proof-of-principle study, which demonstrated the feasibility of an [^18^F]FGln/[^18^F]FDG protocol that leveraged the high sensitivity of LAFOV PET to inject the first tracer at a low dose and the second tracer at a greater dose, overwhelming the signal from the first tracer.

## DISCLOSURE

This work was funded by grants from the National Institutes of Health (R01-CA211337, R01-CA113941), a Cancer Moonshot grant (R33-CA225310), an RSNA Scholar grant (RSCH24‐252), and a grant from the Susan G. Komen Foundation (SAC232145). Daniel Kwon was supported by a UBC MD/PhD Student Scholarship and reports consulting fees from α-9 Theranostics. Austin Pantel reports consulting fees from GE HealthCare. No other potential conflict of interest relevant to this article was reported.

## Notes

KEY POINTS **QUESTION:** Can 2 [^18^F]-labeled radiotracers be imaged in succession in a single-imaging session with LAFOV PET? **PERTINENT FINDINGS:** [^18^F]FGln and FDG were imaged in succession using a low-dose/high- dose protocol on the LAFOV PennPET Explorer with minimal confounding signal from [^18^F]FGln in the [^18^F]FDG image. **IMPLICATIONS FOR PATIENT CARE:** This dual-tracer protocol developed has translational potential, including translation to other novel tracer pairs.
